# Biodiversity and relative abundance of benthic invertebrates in the intertidal of Taraba Sound, Magellan Region, Chile

**DOI:** 10.3897/BDJ.12.e133037

**Published:** 2024-11-27

**Authors:** Ilia Cari, Claudia Andrade, Cristian Aldea

**Affiliations:** 1 Departamento de Oceanografía y Medio Ambiente (DOMA), Instituto de Fomento Pesquero (IFOP), Valparaíso, Chile Departamento de Oceanografía y Medio Ambiente (DOMA), Instituto de Fomento Pesquero (IFOP) Valparaíso Chile; 2 Laboratorio de Ecología Funcional, Instituto de la Patagonia, Universidad de Magallanes, Punta Arenas, Chile Laboratorio de Ecología Funcional, Instituto de la Patagonia, Universidad de Magallanes Punta Arenas Chile; 3 Departamento de Ciencias y Recursos Naturales, Universidad de Magallanes, Punta Arenas, Chile Departamento de Ciencias y Recursos Naturales, Universidad de Magallanes Punta Arenas Chile; 4 Centro de Investigación Gaia-Antártica, Universidad de Magallanes, Punta Arenas, Chile Centro de Investigación Gaia-Antártica, Universidad de Magallanes Punta Arenas Chile

**Keywords:** benthic biodiversity, intertidal species, bivalves, crustaceans, Chilean fjords

## Abstract

The Taraba Sound is situated north of the Strait of Magellan within the Chilean fjord and channel system. In this inlet, samples were collected at three beach levels (supralittoral, mesolittoral and infralittoral) along four transects perpendicular to the coastline. A total of 2,754 specimens were collected and identified, representing seven phyla and 24 operational taxonomic units (OTUs). The dominant components across the four beaches were bivalves and crustaceans, with *Choromytiluschorus* and Paramoeracf.fissicauda contributing significantly to zone similarity. Beaches 2 and 4, located adjacent to river mouths, had lower salinity and consequently lower species richness, abundance, diversity and evenness, particularly in the supra- and mesolittoral zones where freshwater influence is more pronounced. Our study represents the first comprehensive characterisation of intertidal benthic invertebrate biodiversity in the region. Its findings provide valuable baseline data for future research, especially considering the anticipated increase in aquaculture concession applications and the establishment of offshore sea farming centres in the region.

## Introduction

The insular or archipelagic system of Chile extends from Chiloé (41°S) to Magellan (56°S) and is composed of a highly heterogeneous continental shelf with numerous islands, channels and fjords, strongly influenced by freshwater runoff from glaciers and intense rainfall (Camus 2001, Aguilera et al. 2019). This southern region is characterised by a unique and distinctive biogeographical system (Pickard 1971, Antezana 1999, Camus 2001) shaped by tectonic and glacial changes, particularly since Pleistocene glaciation during the Last Glacial Maximum (LGM; Hulton et al. (2002)), which moulded the geomorphology of the channels and fjords throughout the Quaternary period (Hulton et al. 2002, Hüne and Ojeda 2012). As a result of these geological forces, shallow marine and coastal habitats have been significantly affected, leading to disruptions in habitat continuity and availability by altering marine species distribution during quaternary glacial cycles (González-Wevar et al. 2012). Presently, salinity and temperature decrease markedly inland through the channels, with salinity levels dropping below 28‰ near the glaciers and increasing to over 32‰ on the western side of the Strait of Magellan (Vargas et al. 2018). These environmental gradients, shaped by historical and ongoing processes, play a crucial role in structuring marine communities and influencing regional ecological functions (e.g. Cari et al. (2020), Torres et al. (2024)).

This ecosystem, once considered pristine, is now subject to a range of anthropogenic impacts, including salmon farming and marine pollution. Despite its vast expanse of 240,000 km^2^ (Pantoja et al. 2011), research in the central Patagonia region has provided limited information to date. Nevertheless, the existing studies reveal that many macrobenthic organisms are found in this area (Fernández et al. 2000, Forcelli 2000, Retamal and Gorny 2001, Ríos et al. 2003, Montiel et al. 2004, Aldea et al. 2011, Valentich Scott et al. 2020, Aldea et al. 2023). This diversity highlights a unique ecosystem that, due to its vast size and challenging accessibility, has not been adequately studied.

In the fjords and channels of southern Chile, the most abundant macrozoobenthos includes polychaetes, arthropods and molluscs (e.g. Ríos et al. (2003)). The taxonomy of marine invertebrates in this region remains uncertain, largely due to the limited number of experts focusing on these groups and the concentration of studies in more accessible, populated areas (Camus 1998, Arntz 1999, Fernández et al. 2000, Montiel et al. 2004, Häussermann and Försterra 2006). This has left many remote locations unexplored (Camus 1998, Camus 2001). The geographic isolation and environmental variability in this region may result in distinctive faunal assemblages, driven by factors such as species’ tolerance ranges and dispersal mechanisms, particularly in molluscs (Camus 2001). Although comprehensive studies on endemicity rates are still lacking, species in this region play crucial roles in maintaining ecosystem functions, particularly through the provision of ecosystem services (e.g. Rozzi et al. (2012), Castilla et al. (2023)). This makes southern Chile an important area for future research on biodiversity and ecological dynamics.

Taraba Sound represents a relatively unexplored intertidal beach ecosystem. The primary investigations near this area have been conducted as part of the Marine Research Cruises to Remote Areas (e.g. Mujica and Medina (2000), Retamal and Arias (2000), Silva and Calvete (2002), Pizarro et al. (2005), Aldea et al. (2023)) which have provided valuable information to the scientific community. Although no intertidal biodiversity records exist for this specific area, several faunal surveys have been conducted in other parts of the Patagonian region. The objective of this study was to contribute new data on species richness, diversity, evenness and the relative abundance of macrozoobenthos communities in this region.

## Material and methods

### Study Area

**Figure 1.**
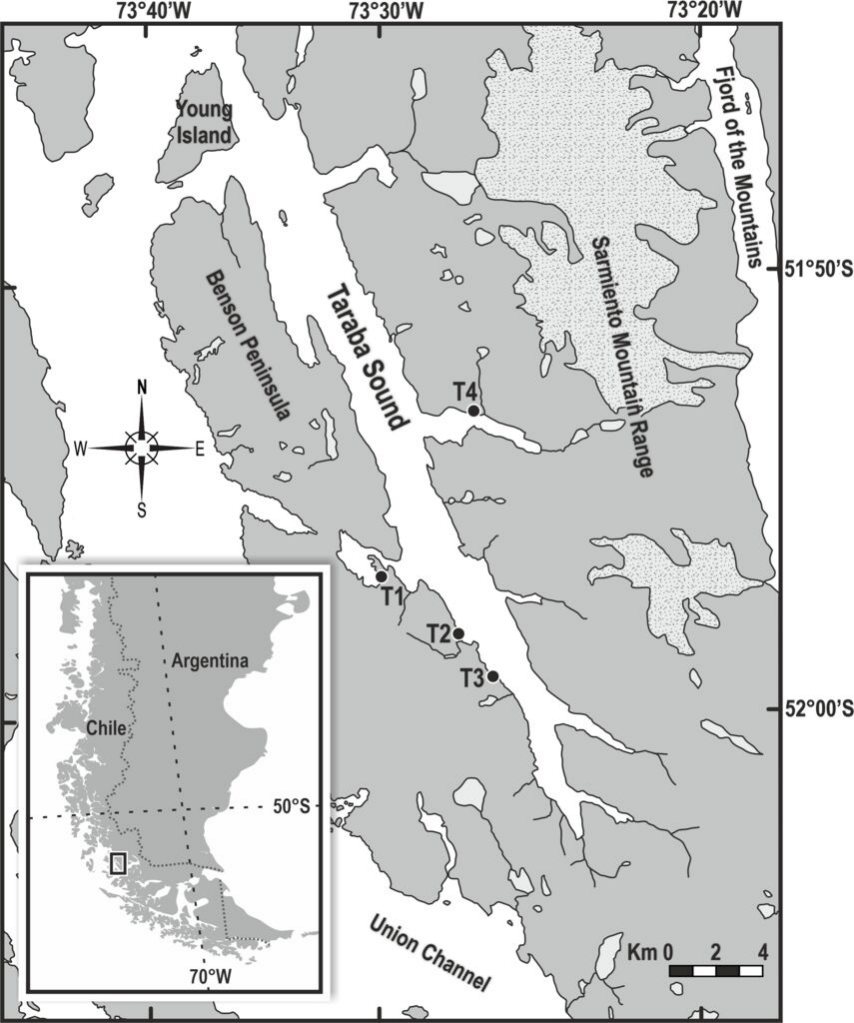
Study area and sampling sites, Taraba Sound.

Taraba Sound is located north of the Magallanes Region (henceforward Magellan Region), the southernmost administrative region of Chile. It spans approximately 31 km in length and is bordered on the east side by the Benson Peninsula and on the west by the Sarmiento Mountain Range, where the Zamudio and Bernal snowdrifts descend into the Fjord of the Mountains (Fig. 1). The interior of the sound is surrounded by mountains and steep rock walls, with temporary freshwater effluents from melting snow on top of the mountains; certain areas are influenced by freshwater runoff from adjacent glaciers. Three main types of environment were observed in the area: strip cliffs, large and rounded cobble fields and gravel and pebble beaches. The last type was used as a protected study site.

The study was carried out on 18 and 19 January 2013 (during the austral summer) on three beaches on the eastern side of the interior of Taraba Sound and one on the west side of the sound. The sampling areas were selected for their easy access during low tide.

### Data collection and sampling

The study areas were accessed using a Zodiac® inflatable boat launched from the fishing vessel *Mary Paz II*. The intertidal benthic macrofauna (> 1 mm) was manually collected and by means of a palette knife to collect sediment between stones directly from the shoreline along transects perpendicular to the coastline on each beach (T1–T4; Table 1). In each transect, samples were taken at specific points determined by the beach profile between high and low tide levels (supralittoral, mesolittoral and infralittoral), N1–N3. In each sampling level, three replicates (R1–R3) were collected using a 0.25 m^2^ quadrat (50 cm x 50 cm) for each beach level. The collected benthic macrofauna was separated, fixed with 5% formalin and preserved in 95% ethanol buffered with saturated sodium borate on board the vessel.

At each sampling site, the geographic coordinates were recorded with an Etrex H® GPS, salinity (‰) was recorded with a handheld refractometer (RHS-10 ATC) and temperature (°C) with a mercury thermometer. In addition, each intertidal sector was photographed using a digital camera (Canon PowerShot A470).

The collected macrofauna was sorted and identified to the lowest possible taxonomic level (i.e. genus or species) starting with specialised literature available for the region (e.g. Forcelli (2000), Zagal and Hermosilla (2007), Häussermann and Försterra (2009)). Some experts assisted in the identification of different taxa (see Acknowledgements). Species records and their respective geographic positions of the sites were entered into a spreadsheet, structured using the Standard Darwin Core format (Wieczorek et al. 2012). The data were submitted in the Integrated Publishing Toolkit, following the GBIF's standards (GBIF: The Global Biodiversity Information Facility 2024).

### Data analysis

The data were analysed using PRIMER-E v.6.0 (Clarke and Gorley 2005). The community structure parameters included total abundance of individuals (N), species richness = number of species (S), Shannon's diversity index = number of species as a function of each species’ abundance (H’; Shannon (1948)), Pielou's evenness index = measurement of how even the abundance of all species is (J’; Pielou (1966)), taxonomic diversity = average taxonomic distance between all pairs of species (∆) and taxonomic distinctness = average path length between two random individuals from different species (∆*), including the taxonomic levels of phylum, class, order, family, genus and species (Warwick and Clarke 1995). To identify groupings or assemblages within areas, we employed non-parametric multivariate analysis techniques using PRIMER-E v.6.0 software (Clarke and Gorley 2005). First, an abundance matrix was developed for the sampling sites and previously transformed using fourth root transformation. To assess biological parameters, the Bray-Curtis similarity index (Bray and Curtis 1957) was then calculated. Following this, to asses other biological parameters, a Non-Metric Multidimensional Scaling (MDS) ordination analysis (Kruskal and Wish 1978) was conducted, along with the creation of a cluster classification dendrogram, based on the average similarity of the sites using the SIMPROF test (Clarke et al. 2008). The similarity percentage (SIMPER) analysis (Clarke 1993) was applied to determine the species contributing most to the observed clusters. Spatial differences in cluster structure were assessed using the one-way ANOSIM non-parametric test (Clarke and Green 1988).

To test for significant differences between study areas (transects) and intertidal levels, a one-way analysis of variance (ANOVA) was performed, followed by post hoc Tukey tests. Before performing these analyses, we verified the normality of the data using the Kolmogorov-Smirnov test with SPSS 15.0 statistical software. A significance level of 0.05 was assumed for the tests to evaluate the null hypothesis.

Pearson correlation coefficients were calculated to explore the relationships between various ecological variables, including species richness, abundance, diversity, uniformity and evenness, in relation to salinity and temperature. Data were analysed using RStudio software (R Core Team 2022), with the ‘ggcorrplot’ and ‘corpmat’ packages employed for visualisation of the correlation matrix via ‘ggplot2’. Coefficients were computed alongside p-values to assess the significance of the correlations. A significance level of p < 0.05 was used to identify the variables most notably related to biological diversity and abundance.

### Repository of material

A data package entitled “Biodiversity of intertidal invertebrates of Taraba Sound (Magellan Region, Chile)” was uploaded to GBIF (Aldea et al. 2024). All material was catalogued with the voucher “intertidal-benthic-invertebrates-taraba-sound2013” and was deposited in the collection of CEQUA Foundation, Punta Arenas city.

## Results

**Figure 2.**
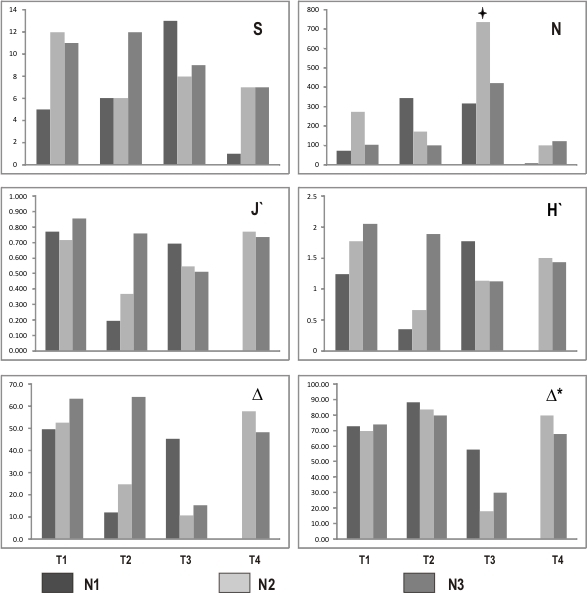
Measures of alpha diversity for different levels and sampling transects in Taraba Sound. N1-3 are low tide levels (supra-meso-infralittoral). T1-T4 are each beach. N: Total abundance of individuals, S: species richness, H': Shannon's diversity index, J': Pielou's evenness index, ∆: taxonomic diversity and ∆*: taxonomic distinctness.

A total of 2,754 specimens were collected and identified, belonging to seven phyla and 24 operational taxonomic units (OTUs). The four beaches were predominantly represented by Bivalvia and Malacostraca, accounting for 95% of the specimens. The highest species richness and abundance were recorded on T3 (S = 17; N = 1473; Fig. 2), while the lowest values were found on T4 (S = 7; N = 225; Fig. 2). The highest values for Shannon's diversity index and Pielou's evenness were observed in the infralittoral zone of the first beach (H’ = 2.058; J’ = 0.858; Fig. 2). Regarding taxonomic diversity, the highest value was observed in the infralittoral zone of T3 (T2N3, ∆ = 64.4) and the highest percentage of taxonomic distinctness was found in the supralittoral zone of T2 (T2N1) at 88.9% (Fig. 2).

The different alpha diversity measures did not show significant differences for any intertidal level (p ≥ 0.05; Table 2). However, significant differences were found amongst the different beaches regarding their abundance (N; p = 0.031; Table 3). Post hoc Tukey tests revealed that the significant differences in abundance were driven by the contrast between beaches T3 and T4 (p = 0.028).

**Figure 3.**
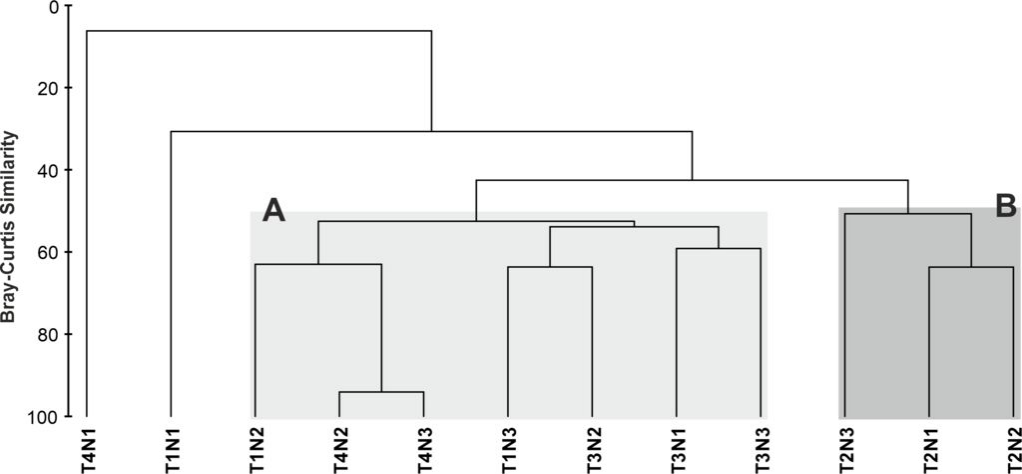
Cluster classification dendrogram of macrofauna similarity in the Taraba Sound. T1-T4 are each beach. N1-3 are low tide levels (supra-meso-infralittoral). A-B represents the different clusters.

The cluster dendrogram found two groups with a similarity level exceeding 50% (Fig. 3). Group A consists of the meso-infralittoral levels of T1 and T4 and all levels of T3. Group B is composed of the three intertidal levels of T2.

**Figure 4.**
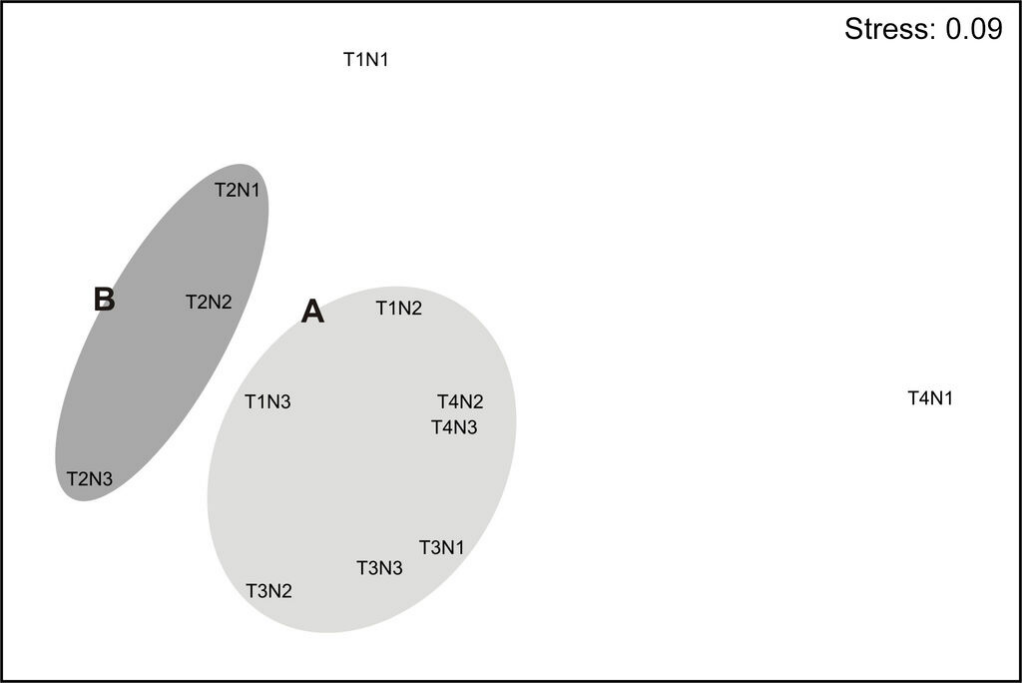
Non-parametric multidimensional scaling analysis (MDS) in the composition of benthic macrofauna in Taraba Sound. T1-T4 are each beach. N1-3 are low tide levels (supra-meso-infralittoral). A-B represents the different clusters.

The non-metric multidimensional ordering (Fig. 4) presents the results observed in the dendrogram, grouping them into assemblages with a Kruskal stress coefficient of 0.09, indicating a reliable representation of the data.

The ANOSIM did not show significant differences in the composition of the intertidal macrofauna between groups A and B (R Global = 0.68; p = 0.80; Table 4).

**Figure 5.**
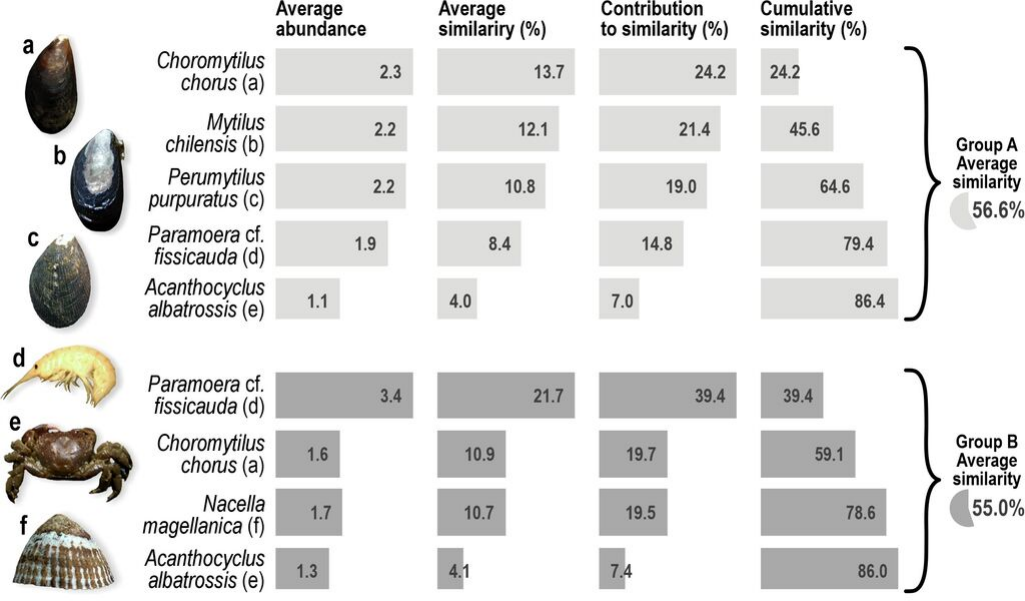
Results of SIMPER similarity percentage analysis of abundance data for species and percentage contribution of different species to the similarity between the studied beaches.

The SIMPER analysis indicated that both groups had very similar internal compositions (Group A 57%, Group B 55%: Fig. 5). The two groups were 57% dissimilar to each other; Group A is mainly composed of the bivalve *Choromytiluschorus* (Molina, 1782) (24%), while Group B is dominated by the amphipod Paramoeracf.fissicauda (Dana, 1852) (39%).

**Figure 6.**
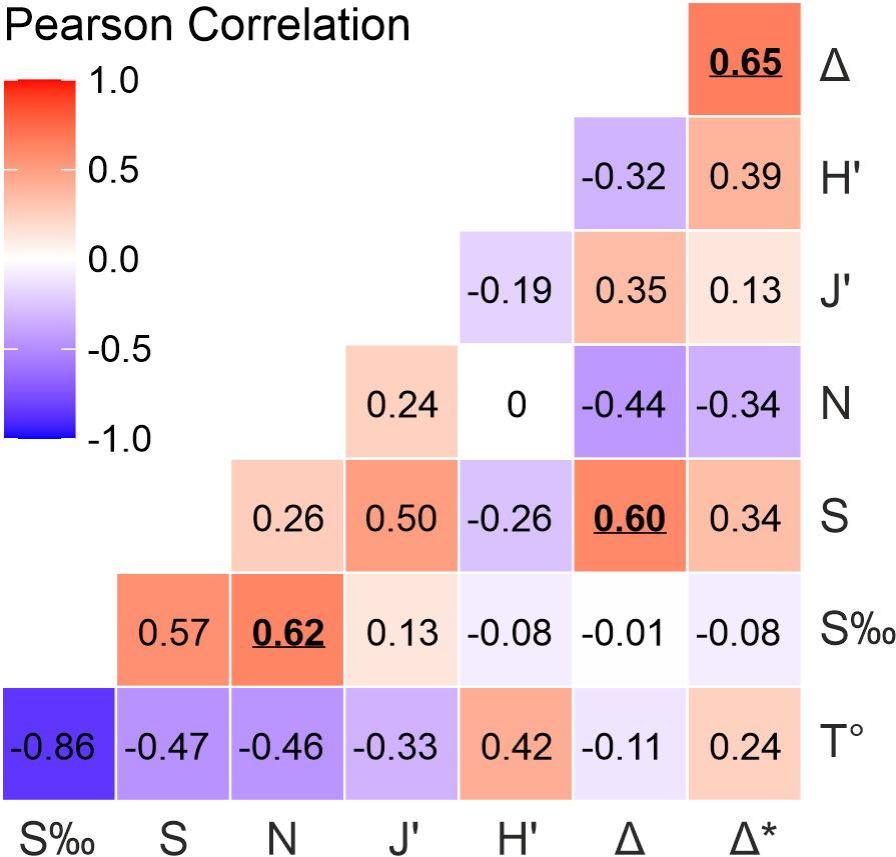
Pearson correlation matrix between measures of alpha diversity and abiotic variables. S (richness), N (abundance) J' (Pielou's evenness index), H' (Shannon's diversity index), Δ (taxonomic diversity), Δ* (taxonomic distinctness), S‰ (salinity) and T° (temperature). Significant values in bold and underlined.

A significant positive correlation was observed between abundance and salinity (R = 0.62, p = 0.03), suggesting that higher salinity values are associated with greater abundance of organisms. Species richness and salinity exhibited a marginally non-significant relationship (R = 0.57, p = 0.053), suggesting a potential trend. Additionally, taxonomic diversity and species richness showed a significant positive correlation (R = 0.60, p = 0.037). Furthermore, a significant correlation between taxonomic diversity and taxonomic distinctness was found (R = 0.65, p = 0.021). All other correlations analysed, including those for abundance, uniformity, evenness, taxonomic diversity and distinctness versus temperature, were not significant at the p < 0.05 level. These results, along with the full range of correlations, provide a visual matrix of Pearson correlation coefficients (R) and highlight the statistically significant relationships (Fig. 6).

## Discussion

Characterising biodiversity and its variability in this vast region is challenging, particularly given that this study depended mainly on the availability of ship time and the weather conditions, which added to the difficulty of studying a very heterogeneous area. For these reasons, it aimed to determine differences between sites. However, a temporal series at each site would have helped define the parameters' variability more precisely and facilitated the comparison of means and tendencies. Since this was impossible, we tried to combine the available data and arrive at conclusions, considering the available literature. This study provides a baseline for future assessments of the environmental impacts of fisheries and other anthropogenic activities. It offers a snapshot view of the current state of the ecosystem. However, it is a necessary first step for monitoring future changes.

The unique geographical features of this southern Chilean area, shaped by various environmental factors, such as tolerance ranges and dispersion processes, make research in this area even more relevant for understanding marine ecology in the Patagonian region. By collecting and documenting biodiversity data in this unique and relatively unexplored ecosystem, this study provides baseline information that can contribute to future research on marine life in the region. Although this work does not directly address resource management, it offers essential foundational data that can support sustainable management and conservation efforts, particularly in the context of ongoing environmental changes and anthropogenic impacts. These efforts align with the goals of Chile's *Plan de Adaptación al Cambio Climático en Pesca y Acuicultura* (PACCPA), a key policy initiative emphasising the need for scientific research to support climate change adaptation in marine ecosystems. The biodiversity data and community structure parameters collected here can serve as a reference for tracking changes over time, supporting ecological monitoring and preserving this ecosystem’s integrity (Aldea et al. 2023).

In the year following this assessment, eight aquaculture concessions were sought from the Chilean Undersecretariat for Fisheries and Aquaculture within the study area (SUBPESCA 2016). To date, seven were granted and six have an application in process. Our study provides critical baseline data to evaluate the effects of these aquaculture activities on the local environment. Reporting these biodiversity records is essential to evaluate the impact of anthropogenic pressure following aquaculture sea-cage installations. Intertidal zones, which serve as refuges, nurseries and feeding grounds, may be vulnerable to changes in water quality, pollution, competition for resources and the introduction of non-native species. Continuous monitoring will be crucial to evaluating habitat changes and their potential consequences on local biodiversity.

### Diversity across beaches

Our study reveals important patterns in the structure of intertidal communities. While we did not observe significant differences in alpha diversity between intertidal levels, we found notable variations in organism abundance across the four pebble beaches studied. Although species richness (S) remains relatively consistent across levels, the composition and abundance of individuals vary, indicating that certain taxonomic groups are more sensitive to environmental factors, such as habitat stability.

The mussel *Choromytiluschorus* dominated the mid-intertidal and infralittoral zones, forming distinct bivalve belts, consistent with the observations of Ríos and Mutschke (1999), where *Mytiluschilensis* dominated in boulder and cobble intertidal areas, also forming distinct bivalve belts in the Magellan Region. In contrast, amphipods were more abundant in the supralittoral zones, likely due to their greater tolerance to desiccation and wave action. All beaches have similar pebble substrates. However, exposure may vary due to differences in habitat orientation and local hydrodynamic conditions, which could affect the abundance of macrozoobenthos communities.

According to Ríos (2007), local factors like wave exposure and wind significantly shape rocky intertidal communities in the Magellan Region. These factors likely influence the patterns we observed, where sessile bivalves dominate in more stable zones, while amphipods thrive in more variable conditions. To better understand how these communities respond to environmental stressors, future studies should explore the functional traits of key species. Such an approach would offer insights into their ecological roles and help predict responses to environmental changes.

Furthermore, a stable species richness combined with a decrease in taxonomic distinctness (Δ*) might suggest a loss of diversity at higher taxonomic levels. Previous studies have shown that the relationship between species richness (S) and taxonomic distinctness (Δ*) can vary significantly across ecosystems, with some showing a positive correlation and others showing no significant relationship or even a negative correlation (Heino et al. 2005). For example, in freshwater ecosystems, S and Δ* respond differently to various environmental gradients, underscoring the importance of using both measures to comprehensively assess community-level biodiversity (Heino et al. 2005). This highlights the need for a multi-faceted approach when examining biodiversity, as richness alone may not fully capture the effects of environmental variability on community structure.

Some studies have reported the importance of the hydrological factors, including salinity gradients within the region that could play a pivotal role in moulding benthic communities in fjord ecosystems (Cari et al. 2020). Particularly noteworthy is the longitudinal gradient observed within inner fjords, where river discharge into the ocean introduces freshwater, profoundly influencing the composition of organisms and the availability of carbon food sources crucial for their sustenance. This salinity gradient delineates distinct ecological niches along the fjord's inner reaches. Our results showed how salinity variation may alter intertidal marine life’s abundance distribution and composition. However, it is important to consider these results with caution, as only one measurement of salinity and temperature was collected per sampling site. This limitation underscores the need for careful analysis and the importance of future research to obtain multiple data points and a more comprehensive understanding of how salinity, temperature and other variables affect biological diversity by collecting additional measurements across the supralittoral, mesolittoral and infralittoral zones.

Organisms dwelling in these transitional zones must probably adapt to fluctuating salinity regimes, affecting their metabolic processes and resource utilisation strategies (e.g. Javanshir (2013), Telesh et al. (2013), Little et al. (2017)). Further research is needed to investigate the potential impacts of salinity variability on species composition, abundance and diversity, as well as to identify which organisms may be more tolerant or sensitive to these changes and their functional consequences.

Despite the fjords' environmental conditions, such as substrate composition, lack of algal associations and low exposure from wave action, our results reveal a remarkable diversity across the four studied beaches, with 24 OTUs identified on intertidal boulders and cobble terraces. This finding falls within the range of variability documented in other studies within the geographic region of the Patagonian fjords. Cari et al. (2020) reported 15 taxa in the inner section of the Baker fjord (47.5°S), while Bahamonde et al. (2022) found 29 taxa in Fjord of the Mountains (52°S; see Fig. 1). Soto et al. (2012) and Soto et al. (2015) reported 30 OTUs across 12 intertidal stations in an extensive area between Concepción Channel (50.1°S) and Smyth Channel (52.7°S) surveyed by the CIMAR 15 Fjords Cruise (CONA 2010, Aldea et al. 2022). Additionally, Ríos and Mutschke (1999) and Ríos (2007) documented 60 OTUs in the Whiteside Channel (54°S), which highlights the variability in biodiversity observed across a similar geographic range. This variability underscores how local environmental factors, such as glacial influence, ocean currents and substrate characteristics, can influence biodiversity and species distribution in these ecosystems, beyond surface conditions. However, there is still a lack of taxonomic knowledge, even though this is necessary for advancing ecological research. Such knowledge is important for proper descriptions of benthic communities, their structure and composition and other comparisons of species richness and diversity in the region, especially taking into account the Patagonian fjord ecosystems in southern Chile as a highly vulnerable region (Iriarte et al. 2010).

In the present study, species richness and taxonomic diversity showed a natural positive correlation, as expected, given that taxonomic diversity is inherently influenced by the number of species present. This relationship is a well-established baseline in biodiversity studies. However, no significant differences were observed between species richness and evenness in our dataset, suggesting that the community is relatively balanced in terms of species distribution or an empirical relationship between species richness and evenness, considering the number of species (Stirling and Wilsey 2001).

### Grouping provided by the cluster

T4 exhibited lower values of species richness and abundance, this trend could be attributed to a lower salinity level (25‰) influenced by freshwater discharges from effluents in the vicinity, originating from a source near the study area (see Fig. 1). Such changes in salinity significantly impact the composition of benthic invertebrate macrofauna, predominantly small bivalve molluscs and amphipod crustaceans, representing three distinct species. Higher salinity environments are associated with greater species diversity, particularly amongst invertebrates (Soto et al. 2012), a pattern observed at higher latitudes as well.

Salinity values in the study ranged from 25 to 30‰, reflecting the influence of nearby freshwater inflow, while a salinity of 35‰ is considered fully marine. Beaches T2 and T4 located near freshwater effluents recorded the lowest salinity values, which might be associated with diminished species richness, abundance, diversity and evenness, especially in the supralittoral and mesolittoral zones. In contrast, beaches T1 and T3 registered higher salinity levels, as they lacked freshwater inflows in their vicinity.

The intertidal fauna of Taraba Sound, overall, inhabits a low-wave energy environment, characterised by semi-protected beaches. This unique setting likely contributes to the specific community structure observed in the region, highlighting the interplay between salinity variations and wave exposure on the biodiversity of these coastal environments.

## Conclusions

This study presents the first record of biodiversity in the remote Taraba Sound. The composition of the four beaches was dominated by bivalves and crustaceans, with *Choromytiluschorus* and Paramoeracf.fissicauda contributing to the highest percentage of similarity of the zones. Other species, such as the bivalves *Mytiluschilensis* and *Perumytiluspurpuratus* and the crustaceans *Acanthocyclusalbatrossis* and *Transorchestia* cf. c*hiliensis* contributed to the formation of the clusters. Salinity levels, which range from 25–35‰, align with the influence of nearby freshwater inputs, indicating a potential gradient that may affect community structure. While the overall diversity in this region may appear low, the distinct species composition and relationship between ecological metrics with environmental variables provide valuable insights into the ecological dynamics at play. Given the remote nature of this area, intensive studies along the fjord and channel belt are essential to better understand the health of coastal communities. Such research can further elucidate the impacts of climate change and anthropogenic pressures resulting from increased salmonid farming, pollution and habitat loss in the region, offering a clearer picture of biodiversity and ecosystem functioning over time.

## Figures and Tables

**Table 1.** Examined sites.

Sampling sites	Latitude (°S)	Longitude (°W)	Temperature at 0 m (°C)	Salinity at 0 m (S‰)
T1	51°56'58.5''	73°30'01.6''	12	33
T2	51°58'25.8''	73°27'34.6''	14	30
T3	51°59'20.4''	73°26'27.8''	12	35
T4	51°53'40.0''	73°27'19.3''	14	25

**Table 2.** Analysis of variance for different measures of alpha diversity amongst the three intertidal levels, covering S (richness), N (abundance) J' (Pielou's evenness index), H' (Shannon's diversity index), Δ (taxonomic diversity) and Δ* (taxonomic distinctness).

**ANOVA one factor**	**S**	**N**	**J**’	**H**’	∆	∆*
**F**	1.007	1.301	1.431	1.728	0.817	0.9
**p**	0.403	0.339	0.289	0.232	0.472	0.914

**Table 3.** Analysis of variance for different measures of alpha diversity amongst the four beaches studied. Bold type indicates significant differences.

**ANOVA one factor**	**S**	**N**	**J**’	**H**’	∆	∆*
**F**	1.301	4.994	0.914	0.847	0.982	2.554
**p**	0.339	0.031	0.476	0.506	0.448	0.129

**Table 4.** ANOSIM test results for abundance of species between groups A and B.

**Factor**	**Level**	**R Global**	**Significance level**	**Nº Permutations**
Cluster	A-B	0.679	0.8	120
